# The Other Side of the Fascia: Visceral Fascia, Part 2

**DOI:** 10.7759/cureus.4632

**Published:** 2019-05-10

**Authors:** Bruno Bordoni, Marta Simonelli, Bruno Morabito

**Affiliations:** 1 Cardiology, Foundation Don Carlo Gnocchi, Milan, ITA; 2 Osteopathy, French-Italian School of Osteopathy, Pisa, ITA; 3 Osteopathy, School of Osteopathic Centre for Research and Studies, Milan, ITA

**Keywords:** fascia, myofascial, smooth muscle, osteopathic, mesoderm

## Abstract

In osteopathic clinical practice and in the teaching of osteopathic medicine, the visceral manipulation approach is included. The knowledge that some viscera satisfy the definition of fascial tissue will allow the osteopath to improve its practice. In the second part of the article, we will give a conclusive definition of fascia, and we will explain the embryological development of the heart and how the fascial tissue can be subject to manual treatment. This text is the first in the international scientific field that discusses the inclusion of some viscera in the context of what is considered fascia, through our committee for the definition and nomenclature of the fascial tissue of the Foundation of Osteopathic Research and Clinical Endorsement (FORCE).

## Introduction and background

In the previous article, we described the viscera with a mesodermal embryological origin, to highlight the presence of fascial tissue (connective tissue, fibroblasts, and smooth muscle cells) on the structure of the bowel. It is clear that the mesoderm, through different growth factors, is fundamental for the development and for the proper functioning of the endoderm and the epithelial tissue [1-4]. The fascial tissue is highly adaptable to the mechanical-metabolic stressors, and this fundamental quality allows the structures that surround, supports connects, protects, and nourishes to survive and evolve [5-8]. Our Foundation of Osteopathic Research and Clinical Endorsement (FORCE), through our committee for the definition and nomenclature of the fascial tissue, in our previous work defined the fascia as follows: “The fascia is any tissue that contains features capable of responding to mechanical stimuli. The fascial continuum is the result of the evolution of the perfect synergy among different tissues, liquids and solids, capable of supporting, dividing, penetrating, feeding and connecting all the districts of the body, from the epidermis to the bone, involving all the functions and organic structures. The continuum constantly transmits and receives mechano-metabolic information that can influence the shape and function of the entire body. These afferent/efferent impulses come from the fascia and the tissues that are not considered as part of the fascia in a bi-univocal mode [9].” In osteopathic clinical practice and in the teaching of osteopathic medicine the visceral manipulation approach is included. Knowing that some viscera satisfy the definition of what fascial tissue is, will allow the osteopath to improve its practice. In this second part, we will describe the embryological development of the heart, we will highlight the literature that demonstrates the effects of visceral osteopathic manual treatment, and we will give a conclusive definition of fascia in order to improve clinical practice.

## Review

Embryological heart development

The mesoderm is divided into three layers: the paraxial mesoderm; the intermediate mesoderm; and the lateral plate mesoderm. The paraxial mesoderm will constitute the somites, from which will derive the axial skeleton, the dermis and the muscles of the back, the thorax and part of the neck. The gonads, kidney, and reproductive tract are derived from intermediate mesoderm. The lateral plate mesoderm splits into parietal (somatic) and visceral (splanchnic) layers: the parietal layer forms the lateral body wall folds and the skeletal striated muscle, and the visceral mesoderm forms the walls of the gut tube [10]. According to other authors, there is a fourth mesodermal layer that will give rise to cardiac tissue: the cardiogenic mesoderm [11]. Cells from the cardiogenic mesoderm will migrate cranially to laterally by enveloping the cranial neural crests; this formation is called the first heart field. The second heart field derives from the anterior lateral plate mesoderm, located medially and caudally to the first heart field [12]. From the anterior lateral plate mesoderm, on both sides, will derive two sets of cells which fuse to form a single primitive heart tube by coalescence; the tube consists of myocardial cells (myocardium) and endothelial cells (endocardium) [11]. An extracellular matrix (ECM) divides the two types of cells. The ECM layer is called cardiac jelly. The epicardium will be formed later by precursor cells or proepicardial cells [11]. The first heart field will form the first part of the heart tube, while the bulk of heart tube lengthening comes from proliferation within the second heart field; the tube has peristaltic movements starting from the 21st day of gestation [11]. Between days 23 and 28, the primitive heart tube elongates unevenly, twisting toward the right and folding, thanks to the endocardial cells, starting to constitute what will be the future morphology of the heart [11]. Between days 24 and 25 the heart starts to pump blood. The new morphology assumes a medial alignment and this change of position is known as convergence. Then a process called septation occurs, that is the division of the primitive ventricle, the aorta and the pulmonary artery, which structures will be linked (phase called wedging) between them through a counter-clockwise movement. Atrial septation begins between the fourth and fifth week of gestation [11]. Between weeks five and eight, the atrioventricular valves form from the endocardium. The ectoderm gives rise to the flaps of the aortic valve and pulmonary vein and will form part of the ventricular septum [11]. From the second heart field derives the cardiomyocyte progenitor cells (CMPCs), smooth muscle cells, fibroblasts, telocytes, and interstitial cells [12]. Multiple signal molecules are indispensable for heart development during gestation: the most important ones are transforming growth factor (TGF), neurogenic locus notch homolog protein (NOTCH), bone morphogenetic proteins (BMPs), and nodal growth differentiation factor (NODAL) [11]. The left ventricle and part of the right and left atrium originate from the first heart field; the right ventricle and part of the right and left atrium originate from the second heart field [11].

Cardiac cells

**Figure 1:**
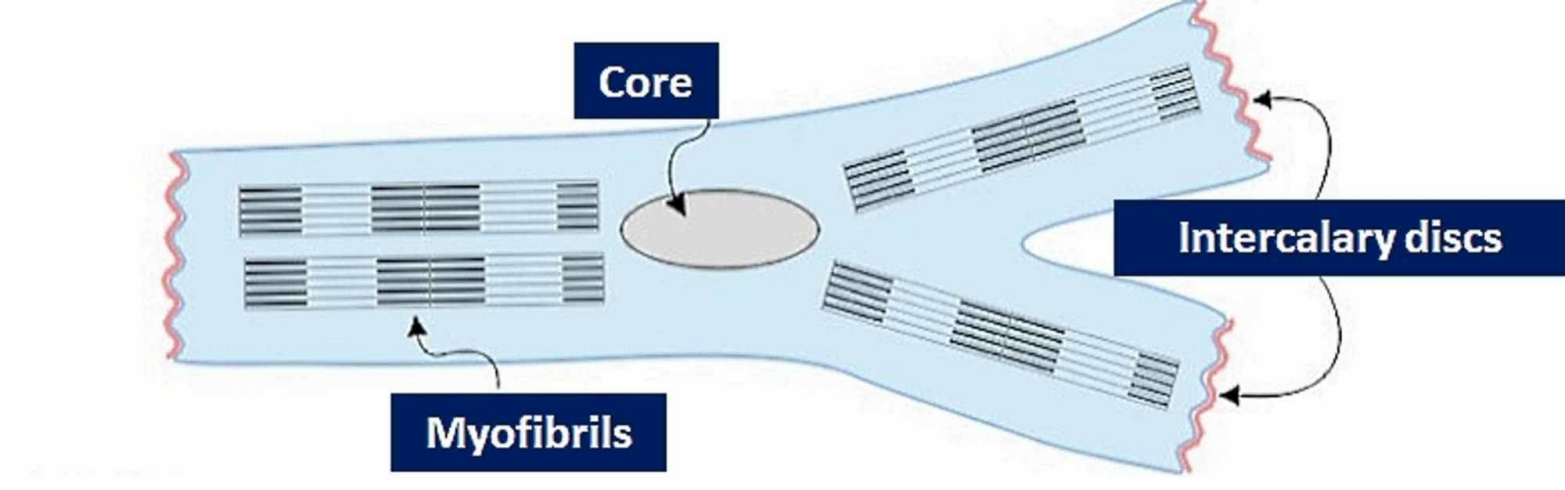
The figure illustrates a cardiomyocyte, with the nucleus, myofibrils and intercalary discs.

A healthy heart properly handles the mechanical tensions it receives to maintain the appropriate shape and function to the body's needs for blood. The cells receive constant deformations due to the heartbeat, the bloodstream, the movement of the diaphragm, and the lungs surrounding the heart; stress can derive from the pericardium, which can be influenced by the esophagus, the descending aorta and its insertion on the sternum, the thoracic vertebrae (D10-D11), the bronchi, and the trachea [13-14]. The cardiomyocyte or involuntary striated muscle cell in a healthy heart is the cell that first manages tensions; the ECM rich in collagen and fibroblasts will vary its density based on the information it will receive from the cardiomyocyte [15]. Myosin is linked to nuclear membrane protein lamina-associated, which deforming the chromatin stimulates the mechanotransduction response; surface myosin (membrane) perceives mechanical variations through integrins [15]. A perfect balance between these cells allows a correct generation and transmission of the electrical signal for heart contraction [15]. During cardiogenesis myocytes can develop into cardiomyocytes or electrical conduction cells; the electrical conduction system of the heart consists of specialized heart muscle cells [16]. The development of the electrical conduction system of the heart begins during gestation and continues throughout life. At the end of cardiomyocytes, there are gap junctions (type 43 and 45 connexins): the latter act as micro-conduits of ionic conduction between the cells. These proteins allow the effective diffusion of the action potential across the cell membrane of heart cells [16]. The electric conduction fibers are held in place thanks to the cardiac connective tissue, increasing the function; connective tissue saves biochemical energy (contraction of cardiomyocytes) through its elastic hysteresis (Figure 1) [17].

Cardiac tissue has also other proteins such as telocytes, sited in all layers of the heart (epicardium, myocardium, endocardium) as interstitial proteins. Probably, besides reinforcing the cardiac structure, telocyte helps the proper electrical conduction in the cardiac layers, facilitating the electromechanical coupling between cardiomyocytes and fibroblasts transporting ions [17]. The cardiac muscle fibers are oriented along different vectors in the epicardium and in the underlying layers; their arrangement and their contractile activation recall the embryological torsional movement in a counterclockwise direction, a movement that is found in the adult heart [18-19]. The cardiac structure is formed by a double helix differently oriented in continuity with atria and ventricles [19]. In the normal heart, the base rotates clockwise during systole and the apex rotates counterclockwise [20-22].

The manual osteopathic approach to viscera

**Figure 2:**
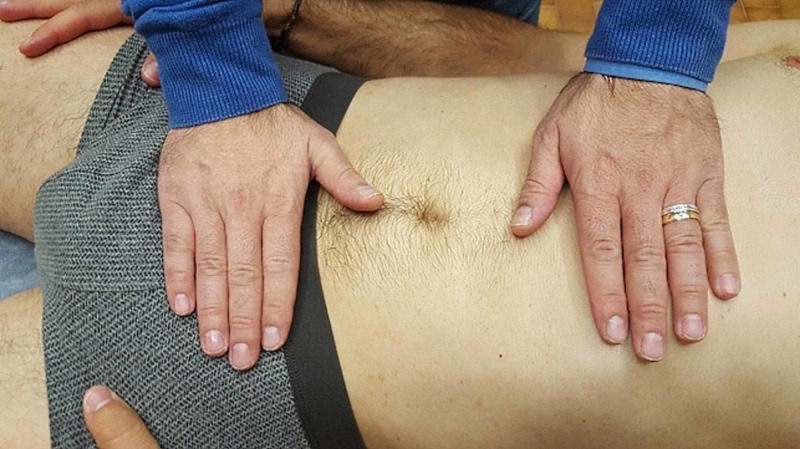
The figure shows a general osteopathic palpation of the viscera of the abdomen.

In the most important book in the world of osteopathic medicine it is described that an organ can be palpated and that one of the goals of osteopathic manual treatment (OMT) is to restore its proper mobility and its optimal function, within the limits allowed by the pathology or by the presence of dysfunction (Figure 2) [23].

Fascial OMT has multiple positive local and systemic clinical results. It improves the response of the parasympathetic system, by decreasing heart rate and breathing rhythm; it improves neuromuscular coordination; it reduces the systemic inflammation indexes (pro-inflammatory cytokines); it improves the interoceptive response; and it raises the pain threshold (medullary and central) [24-27]. The fascial OMT reduces the hospital length of stay and allows to reach early clinical stability [28-31]. The OMT can influence not only the tissue but the single cell, changing the morphology and the fibroblast vectors [32-37]. Visceral mesoderm falls within the definition of fascia. There are several articles in literature that explain how the OMT fascial can improve the function of the organ, the symptoms, the quality of life in case of chronic visceral disorders, using manual indirect techniques on the organ (through pressure on the different tissue layers to influence the bowel): in some cases there is a reduction in drug dosage for pain and the reduction of pain in somatic areas (viscerosomatic reflexes) [38-45]. Further research is needed to provide evidence of the effectiveness of the visceral approach [46]. Viscera can be palpated and treated with positive results in small studies or in case reports. Furthermore, we believe that this article could be a stimulus for OMT researches in the visceral field, considering that in this article the viscera are part of the fascia. The main objective of the osteopathic approach is to improve the movement between the different fascial structures and at different depths, to allow an adequate movement of liquids (blood and lymph are fasciae). This activates the mechano-metabolic responses of the tissues and osteopathy begins at that moment [8]. The fascial and visceral OMT allows involving not only a specific tissue or area but everything that is considered fascia, regardless of where the osteopath's hands are applied [47].

Definition of fascia

At the end of the second part of this article, we have enough elements to give a new definition of fascia. Drawing the attention to our recent definition, we have added new tissues that should be compared to what fascial tissue is: “The fascia is any tissue that contains features capable of responding to mechanical stimuli. The fascial continuum is the result of the evolution of the perfect synergy among different tissues, liquids and solids, capable of supporting, dividing, penetrating, feeding, and connecting all the districts of the body: epidermis, dermis, fat, blood, lymph, blood and lymphatic vessels, tissue covering the nervous filaments (endoneurium, perineurium, epineurium), voluntary striated muscle fibers and the tissue covering and permeating it (epimysium, perimysium, endomysium), ligaments, tendons, aponeurosis, cartilage, bones, meninges, involuntary striated musculature and involuntary smooth muscle (all viscera derived from the mesoderm), tongue. The continuum constantly transmits and receives mechano-metabolic information that can influence the shape and function of the entire body. These afferent/efferent impulses come from the fascia and the tissues that are not considered as part of the fascia in a bi-univocal mode.”

## Conclusions

This second part discussed the inclusion of some viscera in the fascial field, through our committee for the definition and nomenclature of the fascial tissue of the FORCE. The article has revised the embryological derivation of the thoracic and abdominal, considering fascia as those tissues of mesodermal derivation. The viscera included are the gastrointestinal tract, gallbladder and ductus choledochus, larynx, bronchi, lungs, heart, spleen, peritoneum, and urogenital apparatus. Furthermore, we believe that this article could be a stimulus for future OMT trials, pilot studies, and case reports in the visceral field, considering that in this article the viscera are considered part of the fascia. It is necessary to strengthen the efficacy and effectiveness of the OMT, with the aim of obtaining new clinical tools for the treatment and care of patients.
